# Evaluation the Effect of Rosmarinic Acid as an Antioxidant Agent on Shear Bond Strength of Resin Composite to Bleached Enamel

**DOI:** 10.4317/jced.62309

**Published:** 2025-06-01

**Authors:** Hamideh Sadat Mohammadipour, Majid Akbari, Moona Zamanpour, Atefeh Nemati-Karimooy, Arsalan Shahri, Mehrzad Khorshid

**Affiliations:** 1 Associate Professor, Dental Materials Research Center, Mashhad University of Medical Sciences, Mashhad, Iran. Department of Restorative Dentistry, School of Dentistry, Mashhad University of Medical Sciences, Mashhad, Iran; 2 Professor, Department of Restorative Dentistry, Dental Research center, School of Dentistry, Mashhad University of Medical Sciences, Mashhad, Iran; 3 Dentist, Mashhad, Iran; 4 Assistant Professor, Department of Restorative and Cosmetic Dentistry, Dental Research center, School of Dentistry, Mashhad University of Medical Sciences, Mashhad, Iran; 5 Dentist, Research Assistant, Dental Materials Research Center, Mashhad Dental School, Mashhad University of Medical Sciences, Mashhad, Iran

## Abstract

**Background:**

Tooth bleaching, a routine esthetic dental procedure, can compromise bond strength to enamel and dentin, especially if composite restorations are bonded immediately post-bleaching due to residual peroxide and free radicals. To address this, various treatments, including antioxidants like sodium ascorbate (SA), have been used, though SA may increase bacterial accumulation. Rosmarinic acid (RA), an alternative antioxidant, offers additional antibacterial and adhesive benefits. This study evaluates the effect of RA application time on shear bond strength (SBS) in bleached enamel, comparing its efficacy with SA under varying treatment durations. Three null hypotheses regarding RA’s impact on SBS are tested.

**Material and Methods:**

The labial surfaces of 60 freshly extracted bovine incisors were randomly assigned into 6 groups and subjected to a specific surface treatment as follows: no bleaching procedure (NBL), bleaching with 38% hydrogen peroxide (BL), BL+ 10% sodium ascorbate for 10 min (SA), BL+ RA for 10 seconds (RA1), 60 seconds (RA2) and 10 min (RA3). The resin composite was bonded to enamels s immediately after these treatments. After storage in water for 24 hours, the bonded samples were mounted on a universal testing machine and loaded to the fracture point. Data analysis was performed using Kolmogorov-Smirnov, Lilliefors, One-way analysis of variance and Games-Howell tests.

**Results:**

The NBL group had the highest SBS, comparable only to SA (*P*= 0.908). SA outperformed all RA groups (*P*< 0.001). RA3 showed the lowest SBS, significantly lower than RA1 (*P*= 0.011). The main fracture mode in all research groups was adhesive failure.

**Conclusions:**

Rosmarinic acid was not able to reverse the bond strength to enamel immediately after the bleaching process, regardless of the duration of application.

** Key words:**Antioxidant agents, Dental bleaching, Enamel, Rosmarinic acid, Shear bond strength.

## Introduction

Tooth bleaching as a conservative treatment have become one of the most important parts of routine esthetic procedures in dental clinics. The bleaching agents, including hydrogen peroxide (H2O2) and carbamide peroxide, however, are known to adversely affect the bond strength of resin materials to dental structures when restorations are immediately performed after bleaching. During the course of bleaching, long-chain pigmented molecules oxidize and break down into smaller and lighter ones with the release of carbon, water and oxygen. The presence of peroxide remnants and free oxygen radicals can interfere with the resin polymerization which results in compromising the bond strength of resin material to bleached dental structures (1). The compromised bond strength poses a significant clinical challenge, necessitating strategies to mitigate this issue.

Different time intervals (24 hours to 4 weeks) have been recommended in the literature for the implementation of bonding procedures after the bleaching. This time interval not only reverses reduced bond strength but also allows for achieving color stability (2). Nevertheless, it increases the number of office visits, thus bringing great inconvenience to patients (3). Several treatment modalities, including the removal of enamel superficial layer (4), alcohol treatment before the adhesive procedures and after bleaching (5), application of adhesives with organic solvents (6,7) and use of antioxidants (2,8-10) are suggested to compensate for the compromised bond strength occurring immediately after performing the bleaching process. The application of antioxidant agents after the bleaching can shorten the time interval required between the bleaching process and restoration by the elimination of reactive oxygen from the dental substrates (2,9).

Several antioxidant agents have been proposed for this purpose, including sodium ascorbate (1,11) and some natural agents such as grape seed extract, green tee extract, banana extract, lycopene, pine bark extract, aloe vera extract and pomegranate peel extract, which some of them have shown promising results (1,12-15). Sodium ascorbate (SA) is a neutral, nontoxic, water soluble, biocompatible and most commonly used antioxidant agent that could compensate the decreased bond strength of bleached enamel even higher than the bond strength of unbleached enamel (2,11,13,16-19). SA can enhance the bond strength to bleached enamel in shorter time compared with natural antioxidant (20). In spite of the various advantages offered by SA, it has been proven that application of SA can result in accumulation of Streptococcus mutants on bleached surfaces (21). This highlights the need for alternative antioxidant agents with comparable efficacy but improved biological profiles.

Rosmarinic acid (RA) ((2R)-2-[[(2E)-3-(3,4-dihydroxyphenyl)-1-oxo-2-propenyl]]oxy-3-(3,4-dihydroxyphenyl) propanoic acid) is known for having an extensive array of biological activities, such as antioxidant, anti-inflammatory, antibacterial, and antiviral properties (22). There are a number of studies investigating the RA antioxidant effects (23-25). The RA consists of p-toluenesulfinic acid sodium salt. Based on the evidence, the use of this substance for 30 seconds can lead to the enhancement of the bond strength of resin adhesives to the dentin treated with NaOCl and might stabilize the resin-dentin interface (26). Moreover, this chemical substance has the ability to speed up the polymerization of resin composites (27). A commercial product containing RA salt (Accel, Sun Medical Co. Ltd., Kyoto, Japan), which was proposed as a pre-treatment agent for adhesive root canal sealers, was reported to reduce or remove the oxidative impact of irrigation with NaOCl (28). Based on previous reports, the use of this agent for a few seconds leads to the enhancement of bond strength to normal and caries-affected dentin treated with NaOCl (26,29). Despite these findings, limited research exists on the effect of RA as an antioxidant agent in the context of bleached enamel, representing a significant research gap.

This *in vitro* study aimed to evaluate the effect of three application durations of Rosmarinic Acid (RA) on the shear bond strength (SBS) of resin composite restorations to enamel bleached with 38% hydrogen peroxide. The novelty of this investigation lies in exploring RA as a potential alternative to SA for improving bond strength to bleached enamel. The findings aim to provide clinically relevant insights into the practical application of RA in restorative dentistry, potentially offering a more effective and biologically favorable solution compared to SA. Three null hypotheses were considered in this investigation:

1- The application of RA does not affect the SBS of resin composite restorations to bleached enamel

2- The different times of application of RA on bleached enamel cannot present a significant difference in the SBS of the enamel

3- There is no significant difference between the effects of RA and SA on the SBS of resin composite restorations to bleached enamel.

By addressing these hypotheses, this study seeks to determine both the basic scientific efficacy of RA in neutralizing residual oxidative species and its potential clinical relevance as a viable alternative to current antioxidant treatments.

## Material and Methods

- Study design

This *in-vitro* study was conducted at the Department of Esthetic and Restorative Dentistry (Mashhad University of Medical Sciences, Mashhad, Iran). The study protocol was approved by the local ethics committee of Mashhad University of Medical Sciences, Iran (IR.MUMD.DENTISTRY.REC.1394.195).

- Sample size calculation

The power of the sample size was calculated by G Power software (version 3.1, Heinrich-Heine Dusseldorf University, Dusseldorf, Germany) with a 95% confidence interval and 80% power. Based on Silva *et al*. (30) study, examining the effect of antioxidant agents on bond strength of resin composite to bleached enamel with 38% hydrogen peroxide, the sample size for each group in this study was calculated to be 7. Considering the possibility of premature failure during the preparation of samples, 10 samples were considered in each group.

- Preparation of enamel samples

This study was carried out on 60 freshly extracted sound bovine incisors. The physical, chemical, and ultrastructural properties of bovine and human enamel have been compared in various studies, revealing similarities in their structure (31). At first, the samples were cleaned with rubber caps and a slurry of pumice and water, afterwards, they were washed using running tap water. Then, they were inspected using a stereomicroscope (Dino lite Pro, Anmo Electronics Corp, Taiwan) under a magnification of ×10 to discard the samples having cracks or developmental and structural defects. The teeth were then maintained in 0.1% thymol solution at ambient temperature to be used for the study. Then, a low-speed water-cooled diamond saw (CNC Machine, Nemo, Karaj, Iran) was employed to remove the anatomical crowns from root at the cement-enamel junction under running water. Each separated crown was embedded in self-cured acrylic resin (Acropars, Marlic Co., Tehran, Iran) with a 3×3 mm² enamel window exposed in the center of the labial surface to standardize the exposed surface area across all samples. In order to prepare the flat surfaces for resin composite bonding and SBS measurement, the surfaces of the enamels were polished in a serial manner by means of 400, 600, and 800 grit silicon carbide papers (Starcke, Hoffman Co, Germany) under cooling water flow. In the next step, the samples were divided into six experimental groups randomly, each with 10 samples. Ten specimens were separated as a negative control group (NBL group) and left without any bleaching treatment (group 1).

- Bleaching procedure 

The remaining 50 specimens were subjected to bleaching with 38% H2O2 (Opalescence Xtra Boost, Ultradent Products Inc., South Jordan, Utah, USA) for three times, each lasting 15 min in the absence of light activation according to the manufacturer’s guidelines. All samples were subsequently washed with distilled water for 1 min and put in five groups according to different antioxidant application protocols. Group 2 served as the positive control group, receiving only bleaching treatment without any antioxidant application. In other experimental groups, two different antioxidant agents, including SA and RA were used to the specimens immediately after the bleaching procedure.

- Application of antioxidant agents

For the purpose of the study, 10% SA hydrogel was produced using the crystalline form of sodium ascorbat salt (Sigma Aldrich, Madrid, Spain) with a molecular weight of 198.11. The prepared hydrogel containing SA was applied on enamel surfaces with micro brushes for 10 min in SA group and ethanolic RA solution containing 100µ M RA was applied for 10 seconds, 60 seconds and 10 minutes in other experimental groups, respectively. These time points also align with clinically feasible application periods, providing insights into the practical utility of RA in chairside restorative procedures. Following the implementation of antioxidant treatment, the surface of the enamel was completely washed with distilled water for 30 seconds and gently air dried. The summary of different treatment protocols was presented in  Table 1.

- Bonding procedure

In the next stage, the exposed enamel surfaces were subjected to 30 seconds of 35% phosphoric acid treatment (Ultraetch, Ultradent Products Inc., South Jordan, USA). Subsequently, they were rinsed with the water for 5 seconds and then air dried to view the frosted appearance. The etched enamel was then treated with an ethanol-based and water-based etch-and rinse adhesive (Adper Single Bond Plus, 3M ESPE, St. Paul, MN, USA) following the manufacturer’s instructions which is considered a 5th generation bonding agent. Two consecutive layers were applied to the etched enamel, and solvent evaporation provided by gentle air blow, followed by light polymerization for 10 seconds using a light curing device (Blue phase 8, Ivoclar Vivadent, Schaan, Liechtenstein) that was set at 650 Mw/cm2 of power density. In each sample, a plastic mold with a height of 3 mm and a diameter of 1.2 mm, which was held perpendicular over the enamel surface was filled by a resin composite (Filtek Z100, 3M ESPE, St. Paul, MN, USA) with A2 shade. The excess composite was removed with a sharp explorer from the periphery of the mold, and was then polymerized for 40 seconds by putting a light guide (Bluephase C8, Ivoclar Vivadent, Schaan, Liechtenstein) on top of each mold. The power density of light cured device was checked at every 10 exposures. The bonded specimens were placed in distilled water for a period of 24 hours in an incubator (Fine Tech, Shin Saeng, Gyeonggi-do, South Korea) at 37°C with 100% humidity.

- Debonding procedure

The plastic molds were separated and carefully removed after 24 hours. The samples were then placed in a universal testing machine (Santam, model STM-20, Tehran, Iran) in order to import the shear force to the adhesive interface till the incidence of the fracture. The cross-head chisel was inserted perpendicular to the enamel-composite interface to load the samples at a speed of 1 mm/min. The calculation of SBS in megapascals (MPa) was accomplished via dividing the load at the failure point (newtons) by the surface area of the enamel-composite bonding (1.13 mm2).

- Fracture analysis

Following the fracture and removal of the specimens from the testing instrument, the fracture sites were observed with a stereomicroscope (Dino lite Pro, Anmo Electronics Corp, Taiwan) at a magnification size of ×20 to facilitate the identification of the bond failure type. The modes of fracture were categorized into three groups: 1) adhesive failure at the border of the resin composite with enamel substrate, 2) cohesive failure within the resin composite or enamel substrate, and 3) mixed failure as a blend of adhesive and cohesive failures.

Adhesive failure occurs when the bond between the adhesive and the substrate (tooth structure or restoration) breaks. This type of failure indicates that the adhesive did not adequately bond to one of the surfaces. Cohesive failure occurs within the adhesive material itself or within the substrate (e.g., dentin or composite). This means that the material has failed due to internal weaknesses rather than at the interface. Mixed failure is a combination of both adhesive and cohesive failures. It occurs when some parts of the bond fail at the interface while others fail within one of the materials.

- Statistical analysis

The normality of data distribution was investigated by means of the Kolmogorov-Smirnov test and Lilliefors statistical analysis. The comparison of the research groups in terms of SBS value was accomplished using the ANOVA and Games-Howell analyses. Statistical significance was considered at 95% confidence interval by SPSS software, version 22 (Statistical Package for Social Sciences, Version 22.0, SPSS Inc, Chicago, Ill, USA).

## Results

The assumption of normality was determined by Kolmogorov-Smirnov test and Lilliefors statistical analysis (*P*> 0.05 for all study groups). The minimum, maximum, mean and SD of SBS of the experimental groups are demonstrated in  Table 2. The results of One-way ANOVA revealed a significant difference among the research groups (*P*< 0.001). Based on Games-Howell analysis, the SBS of NBL group (negative control group) was significantly higher than other groups (BL and three RA groups), except for SA group (*P*= 0.908). The NBL group showed noticeably greater SBS values than BL group (*P*= 0.24). No significant difference was observed between BL and RA treated groups (*P*> 0.05). The SBS of SA group was significantly higher than the three RA-treated groups (*P*< 0.001) and BL group (*P*= 0.047). The least SBS among the study groups was related to RA3 group and it showed significantly lower SBS compared with RA1 (*P*= 0.011).

**Figure 1:**
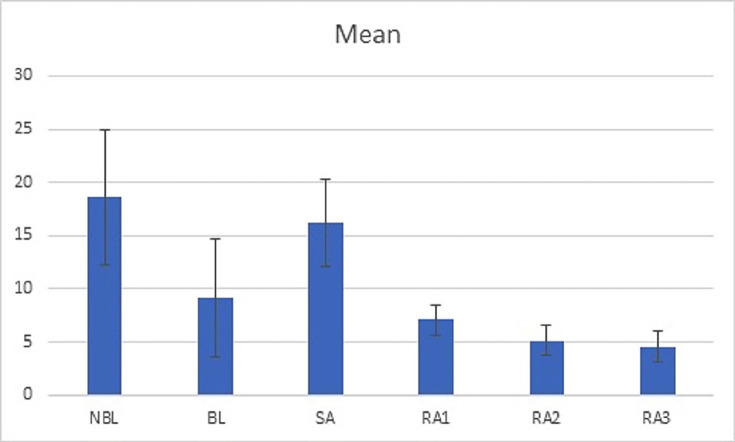
The bar chart presented the comparison of mean, SD, min and max values of bond strength among the different study groups.

The main fracture mode in all research groups was identified as the adhesive failure, (Fig. 1).

## Discussion

In this study, the effect of RA as an antioxidizing/reducing agent on SBS of a resin composite to hydrogen peroxide-treated bovine enamel was evaluated. Based on the obtained results, RA could not improve the SBS of the bleached enamel as SA and there was no significant difference between three times of application of RA.

In this study, the bovine teeth were utilized to assess the SBS of resin composite to bleached enamel. They provide some advantages such as being available, free of caries and have large surface area which facilitates application of more than one specimen (13). Despite the differences of human and bovine enamel in terms of density and porosity, bovine teeth produce comparable results with human teeth on evaluating different surface treatments on dental structures (32).

The same as previous literature that reported deteriorative changes in adhesion to tooth structure after bleaching (15,18) in the current investigation, the SBS of bleached enamel was significantly lower than the sound enamel group (*P*=0.02). Free radicals as molecules with one unpaired electron and high reactivity, which released from the bleaching agents, are able to disintegrate large pigmented molecules into minor and lighter ones. Peroxide or oxygen remnants reduce the adhesion through two main mechanisms. They act through intervening in the infiltration of resin into the etched enamel or inhibition of resin polymerization reaction (32). The SEM images from resin-bleached enamel interfaces showed large areas free of resin. Also, the retained peroxide in the subsurface layer of the enamel, produced the coarse and porous appearance resulted from bubbling from oxidizing reactions (33). This layer and dentinal fluid may be considered as a reservoir for peroxide components that maintain higher than normal levels of peroxide in the bonding interface, thereby leading to the inhibition of polymerization reaction and a reduction of SBS. They could be persistent till being detached via pulpal microcirculation and diffused from the external surface (34). Therefore, the adhesion of dental composite to enamel is compromised by the diminution of the resin tags number and quality (16).

A 7-day interval from bleaching is suggested to remove the residual oxygen from the bleaching material for the aim of obtaining sufficient SBS for clinical conditions (16). The application of antioxidant agents on bleached enamel and dentin can replace delayed bonding. They restore the altered redox potential of the oxidized bonding substrates and allow free-radical polymerization of the adhesives to progress without premature termination leading to reversal of the compromised bonding (11). However, their application may produce some esthetic challenges regarding the stability of color. For esthetic purposes, the final restorations must be postponed for a minimum of 14 days after the implementation of vital bleaching in order to color stability was obtained (32).

As this study finding revealed, SA significantly increased the SBS of resin composite materials that immediately bonded to bleached enamel (*P*=0.047) with no significant difference with the sound unbleached enamel group (*P*=0.908). In agreement with the current research, several previous investigations have indicated the ability of 10% SA to reverse the decreased SBS of bleached enamel the same as the control unbleached samples or even produced higher bond strength (2,11,16-18,31). Indeed, the SA leads to the polymerization of the free radicals of adhesive resin to continue without premature termination, thereby reversing the compromised bonding (17). The SA application might substitute the delay time between enamel bleaching and bonding process (2,16-18). Given the confirmation of the effectiveness of 10% SA for 10 minutes on improving the SBS of bleached enamel in the majority of recent studies (1,11,16,32,34) in the current research the same time and antioxidant concentration were applied. It has been shown SA can neutralize and reverse the oxidative effect of the NaOCl, used as an irrigation solution during endodontic treatment, and enhance the reduced dentin SBS (25,26).

In contrast to the current study, a previous study carried out by Prasansuttiporn *et al*. (25) could not present a significant enhancement in the SBS of adhesives to dentin after SA treatment. This discrepancy can be due to the difference in application time (5 or 10 seconds compared with 10 min in the current study), substrate (dentin versus enamel), and application form of SA (solution instead of gel form). In agreement, a recent study concluded the 10% SA pretreatment may not be suitable for restoring the SBS, since the SA treated group has significantly lower SBS values compared with the non-bleaching one (20). The authors attributed this different outcome to four application cycles of 35% HP used in their study and resulted in higher residual free-radical species compared with the other studies, which used carbimide peroxide and HP in lower concentration and lesser time.

Regarding the outcome of this study, RA could not compensate the negative effect of the bleaching agents on enamel. Indeed, no significant difference detected on SBS of bleached enamel (BL) and RA treated groups (*P*>0.05). Therefore, the authors could not reject the first null hypothesis of this study. The RA is regarded as a diphenolic compound which has two catechol (1,2-dihydroxybenzene) rings. The rings are responsible for the antioxidant activity of RA by forming an intermolecular hydrogen bond between the free hydrogen of its hydroxyl and phenoxyl radicals (24). In contrast with the current research, Taniguchi *et al*. (26) found an increase in µTBS of self-etch adhesives after NaOCl treatment with applying a commercial product containing p-Toluenesulfinic acid sodium salt, ethanol and water (Accel, Sun Medical Co. Ltd., Kyoto, Japan) on caries-affected dentin. The difference in the results may be due to the use of different substrate, the fracture test, and the type of solution containing RA.

Although some studies show that the higher bond strength of the composite to enamel obtain with extended application time of an antioxidant (3,32) in this study the SBS gradually decreased by increasing the application time of RA from 10 seconds to 10 minutes. It means, the SBS of the group treated with RA for 10 seconds was significantly greater than 10 min (*P*=0.011). Thus, the second hypothesis regarding to the difference between three application times of the RA on bleached enamel could be rejected. The authors attribute this outcome to producing the RA salts that cover the enamel surface. Increasing the application time of RA on the surface, the chemical reaction develops and produces salts which do not easily wash with running water. These salts interfere with resin penetration and inhibit resin tag formation that finally compromise the SBS. Conversely, in Kaya *et al*. (32) study, when the application time of SA enhanced from 10 min to 240 min, the bond strength increased. It might be attributed to the gel form of SA that they used. The gel form of SA has a significantly slower release rate and inferior bond strength to the bleached enamel in comparison with the solution form (35). As the gel form needs a long time in order to be effective, it is clinically unsuitable, unless patients apply the antioxidants containing gel in a bleaching tray before restoration treatment (18). Also, in contrast to our results, another study showed that the application of RA was able to improve the immediate bond strength of a 1-self etch adhesive to NaOCl treated artificial caries-affected dentine (CAD) (36). Furthermore, when RA was applied alone the µTBS was significantly enhanced compared to NaOCl treated and untreated artificial CAD. This again may be attributed to the different substrate. While the mentioned study was done on dentine, our study was done on enamel which have different structures.

In the present study, a synthetic powder of SA was prepared and used in the form of 10% hydrogel and RA extract was produced from the rosemary plant in the form of 10% hydroalcoholic extract. Since this material is not soluble in water, necessitating the use of a low concentration of alcohol (5% ethanol) to produce the hydroalcoholic form.

According to an investigation carried out by Prasansuttiporn *et al*. (25), the 5-10 seconds administration of 100 µM RA in 5% ethanol solution results in the improvement of SBS to the dentin treated with NaOCl; however, the application of 10% SA within the same time period does not render similar outcomes. The authors related it to the higher total antioxidant capacity of RA than that of SA, which needs lower application time to increase the SBS to NaOCl treated dentin (27). Moreover, the salt of p-toluenesulfinic acid can efficiently accelerate resin monomer polymerization and lead to a higher degree of conversion in resin polymerization, an outcome that did not confirm by the present study result.

In agreement with the majority of previous studies which indicated the herbal antioxidant had lower bond strength than SA (1,3,13,19) in the present study, RA treated groups showed significantly lower SBS values than SA treated group (*P*<0.001). This result rejects the third hypothesis. The lower SBS in the samples treated with RA comparing with SA may be attributed to differences in water solubility and molecular weight of both chemical agents. The water solubility property of SA facilitates its rinsing from the affected surface without any remnants of the chemical agent (23). In addition, lower molecular weight of SA (176/124 g/mol) compared with RA (360/318 g/mol) can facilitate its penetration within the enamel prisms. Ultimately, the adherence of undissolved RA to the surface of enamel inhibits the role played by antioxidants, thus hindering subsequent bonding.

In Sunnetha *et al*. study (37) the both SA and RA could efficiently regain SBS immediately after the bleaching. This difference might be related to greater concentration of RA, continues refreshing of RA solution, effective removing of residual antioxidant and formed salts from the enamel surface and lower concentration of the bleaching agent in their study (10% carbamide peroxide).

There were controversial reports regarding the effect of RA on dentin substrates after the oxidizing procedures. In contrast with our results, the findings obtained by Khoroushi *et al*. (24) on hydrogen peroxide-treated glass fiber posts which were cemented with self-adhesive cement revealed that application of RA antioxidant improves the SBS in comparison with applying 6.5% Hespridin and 10% SA. The difference in the results of this study with ours may be attributed the different substrate and application time of 20 min of RA on H2O2 treated posts compared with lower times in this research. The other investigation by Khoroush and co-workers (23) on the impact of various antioxidant agents on the SBS of resin cements to NaOCl treated dentin of root canal, was completely in agreement with the present study. Among three antioxidants tested, including 10% RA, 10% hesperidin and 10% SA hydrogel, SA could more effectively regain the debilitated SBS to root dentin after irrigation with NaOCl as high as the negative control group. The authors reported no significant differences between the positive control and RA groups. However, there were differences between their study and the current research. In mentioned study, RA was applied for 2 minutes and there was a one-week delay of bond strength test after NaOCl treatment.

The main limitation of this study was that it was performed *in vitro*, and therefore may not fully reflect the clinical and oral condition. Moreover, this study was only investigated SBS at 24 hours and did not examine the long-term SBS. Due to lack of strong evidence, to determine the effectiveness of RA on recovering the adhesion to compromised bleached dentin and enamel substrates, further laboratory studies should be done. To improve the antioxidant potential of RA in reversing bond strength after the bleaching, it can be suggested to increase the concentration of RA in future studies. In addition, factors like thermal and load cycling and water storage aging methods that challenge the adhesive interface should be considered in further studies when the effectiveness of antioxidant agents on bond quality of resin materials on the bleached tooth structure has been evaluated.

Regarding the limitations of this in-vitro study, the Rosmarinic acid was not able to reverse the bond strength to enamel immediately after the bleaching process, regardless of the duration of application. Applying 10% sodium ascorbate for 10 minutes reversed the compromised bond strength of the bleached enamel as high as the control unbleached values.

## Figures and Tables

**Table 1:** Experimental groups.

Groups	Description
NBL (Negative control group)	No bleaching+ immediate restorative procedures
BL (Positive control group)	Bleaching + immediate restorative procedures
SA	Bleaching + 10% sodium ascorbate + immediate restorative procedures
RA1	Bleaching + 100µ M rosmarinic acid containing solution for 10 seconds + immediate restorative procedures
RA2	Bleaching + 100µ M rosmarinic acid containing solution for 60 seconds + immediate restorative procedures
RA3	Bleaching + 100µ M rosmarinic acid containing solution for 10 minutes + immediate restorative procedures

**Table 2:** Mean, standard deviation (SD), minimum and maximum of SBS of study groups.

Study groups	N	Mean (Pa)±SD	Min	Max	One way analysis of variance
NBL (negative control group)	10	18.59± 6.34^a^^□^	9.63	29.51	F=22.18 P<0.001
BL (positive control group)	10	9.13± 5.54^b^	2.59	17.14	
SA	10	16.18± 4.08^a^	11.75	23.00	
RA1	10	7.05± 1.42^bc^	5.77	9.71	
RA2	10	5.31± 1.44^b^	3.89	8.68	
RA3	10	4.54± 1.43^bd^	2.07	6.28	

□ Means with the same superscript letters are not statistically significant at *P* _>0.05.

## Data Availability

The datasets used and/or analyzed during the current study are available from the corresponding author.
